# Measurement of Differential Na^+^ Eﬄux from Apical and Bulk Root Zones of Intact Barley and *Arabidopsis* Plants

**DOI:** 10.3389/fpls.2016.00272

**Published:** 2016-03-08

**Authors:** Ahmed M. Hamam, Dev T. Britto, Rubens Flam-Shepherd, Herbert J. Kronzucker

**Affiliations:** Canadian Centre for World Hunger Research, University of TorontoToronto, ON, Canada

**Keywords:** *Arabidopsis thaliana*, barley, compartmental analysis, distal root, eﬄux, salinity stress, sodium, SOS1

## Abstract

Rapid sodium cycling across the plasma membrane of root cells is widely thought to be associated with Na^+^ toxicity in plants. However, the eﬄux component of this cycling is not well understood. Eﬄux of Na^+^ from root cells is believed to be mediated by Salt Overly-Sensitive-1, although expression of this Na^+^/H^+^ antiporter has been localized to the vascular tissue and root meristem. Here, we used a chambered cuvette system in which the distal root of intact salinized barley and *Arabidopsis thaliana* plants (wild-type and *sos1*) were isolated from the bulk of the root by a silicone-acrylic barrier, so that we could compare patterns of ^24^Na^+^ eﬄux in these two regions of root. In barley, steady-state release of ^24^Na^+^ was about four times higher from the distal root than from the bulk roots. In the distal root, ^24^Na^+^ release was pronouncedly decreased by elevated pH (9.2), while the bulk-root release was not significantly affected. In *A. thaliana*, tracer eﬄux was about three times higher from the wild-type distal root than from the wild-type bulk root and also three to four times higher than both distal- and bulk-root fluxes of *Atsos1* mutants. Elevated pH also greatly reduced the eﬄux from wild-type roots. These findings support a significant role of SOS1-mediated Na^+^ eﬄux in the distal root, but not in the bulk root.

## Introduction

The entry of Na^+^ into plant cells is considered to be central to Na^+^ stress and toxicity, in large part due to its interference with cytosolic K^+^ homeostasis (Maathuis and Amtmann, 1999; Munns and Tester, 2008). Under saline (sodic) conditions, roots of many plant species appear to have little control over the thermodynamically passive, unidirectional influx of Na^+^ across the plasma membrane; this is suggested by the exceedingly high flux values that are frequently reported for this ion when it is present at high external concentrations (e.g., Lazof and Cheeseman, 1986; Davenport and Tester, 2000; Essah et al., 2003; Malagoli et al., 2008; Wang et al., 2009; Kronzucker and Britto, 2011). To counteract such large influxes of Na^+^, and thus keep cytosolic Na^+^ concentrations low (Carden et al., 2001), a powerful Na^+^ eﬄux system is thought to operate in the plasma membrane of root cells, mediating the extrusion of Na^+^ (Malagoli et al., 2008). This occurs against the ion’s electrochemical potential gradient (Munns and Tester, 2008), and, in conjunction with a poorly restricted Na^+^ influx, results in a rapid cycling of Na^+^ across the plasma membrane. In a recent comprehensive review of Na^+^ eﬄux from roots of higher plants, we have referred to this cycling phenomenon as “Rapid Transmembrane Sodium Cycling,” or RTSC (Britto and Kronzucker, 2015).

The unidirectional flux processes underlying RTSC, however, are poorly understood, and the eﬄux step in particular has not been well characterized even in important model systems such as barley and *Arabidopsis thaliana* (see Britto and Kronzucker, 2015, for review). Nevertheless, it appears likely that Na^+^ eﬄux from roots is at least partially mediated by the “Salt Overly-Sensitive-1” (SOS1) protein, which has been demonstrated to have Na^+^/H^+^ antiport activity in plasma membrane vesicles (Qiu et al., 2002) and to be critical for salt tolerance in many species (Kronzucker and Britto, 2011). Despite these findings, problems regarding the *in vivo* function of SOS1 persist (Ding and Zhu, 1997; Garciadeblás et al., 2007; Oh et al., 2009; Kronzucker and Britto, 2011; Britto and Kronzucker, 2015). One such problem is that the expression of SOS1 appears to be restricted to two specific regions of the root: (1) the xylem parenchyma, where it is believed to play a role in long-distance Na^+^ transport in plants; and (2) in epidermal cells surrounding the meristematic region of the distal root, where it is believed to protect meristematic cells, which lack large vacuoles and thus have a limited capacity to safely sequester Na^+^ (Shi et al., 2002; Oh et al., 2009; Yadav et al., 2012). Corroborating evidence for this localization comes from: (1) the similar expression pattern of RCD1, a protein known to interact with SOS1 in the context of oxidative stress tolerance (Katiyar-Agarwal et al., 2006); and (2) the onset of salt stress playing out in the distal root *sos1*-mutant plants of an *Arabidopsis* relative, *Thellungiella salsuginea* (Oh et al., 2009).

Remarkably, very few examinations of unidirectional Na^+^ eﬄux from plant roots have been made in the context of the RTSC model, particularly under steady-state conditions (most importantly, conditions of constant external Na^+^ supply) and with respect to the functioning and localization of SOS1. Therefore, in the present work, we sought to address this situation, and provide a better means of evaluating the RTSC model and the function of SOS1 *in vivo*. To do so, we developed a chambered cuvette system to isolate regions of the root system from each other, in order to compare the unidirectional eﬄux of ^24^Na^+^ from the distal root and that from the remainder of the root (hereafter called the “bulk root”), in barley and in wild-type and *sos1*-mutated *A. thaliana*. More specifically, we sought to test the hypothesis that Na^+^ eﬄux from roots may be much greater in the distal root than in the bulk root, in accordance with expression patterns of SOS1.

## Materials and Methods

### Plant Culture

Seeds of *Arabidopsis* (*Arabidopsis thaliana* L.) wild-type (ecotype Col-0 *gl1*) and *sos1* (SOS1 loss-of-function mutant) were hydrated in dH_2_O for 30 min, surface-sterilized with 70% ethanol for 5 min, followed by a 10 min wash in a 10% bleach and 0.05% SDS mixture, then rinsed at least three times for 5 min in dH_2_O, and allowed to stratify in 0.1% agar for 3 days in the dark at 4°C. Seedlings were grown in 14-L hydroponic vessels for 5 weeks in climate-controlled growth chambers, with an irradiation of 150 μmol photons m^-2^ s^-1^ at plant height for 12 h d^-1^ (Philips F96T8/TL841/HO/PLUS; Philips Electronics). Day/night temperatures were 20°C and 15°C, respectively, and relative humidity was approximately 70%. The nutrient solution contained 1 mM NH_4_NO_3_, 1 mM KH_2_PO_4_, 0.5 mM MgSO_4_, 0.25 mM CaSO_4_, 25 μM H_3_BO_3_, 20 μM FeEDTA, 2 μM MnSO_4_, 2 μM ZnSO_4_, 0.5 μM CuSO_4_, and 0.5 μM Na_2_MoO_4_. The pH of the solution was set to 6.0 using KOH. Solutions were exchanged weekly. 72 h prior to experimentation, seedlings were transferred to a nutrient solution identical the above, except that it was supplemented with 10 mM NaCl to enhance the expression of SOS1 (Shi et al., 2000).

Seeds of barley (*Hordeum vulgare* L.) were surfaced-sterilized in a 1% bleach solution for 15 min, then washed four to five times with dH_2_O every 20 min. Seedlings were germinated in acid-washed sand for 3 days before transferring them into 14-L aerated vessels containing nutrient solution (modified Johnson’s solution) composed of: 1 mM Ca(NO_3_)_2_, 0.5 mM KH_2_PO_4_, 0.25 mM MgSO_4_, 0.1 mM K_2_SO_4_, 25 μM H_3_BO_3_, 20 μM FeEDTA, 2 μM ZnSO_4_, 0.5 μM CuSO_4_, 0.5 μM MnSO_4_, 0.125 μM Na_2_MoO_4_, and supplemented with 100 mM NaCl to enhance the expression of SOS1 (Chen et al., 2015). The external concentration of Na^+^ was much higher than that used with *Arabidopsis*, reflecting the greater salt tolerance of barley. The pH of the solution was set to 6.3–6.5 using KOH. Solutions were completely exchanged on day five following germination to ensure the nutritional steady state for experimentation on day seven. Seedlings were grown in climate-controlled chambers with an irradiation of 200 μmol photons m^-2^ s^-1^ at plant height for 16 h d^-1^ (Philips Silhouette High Output F54T5/850HO; Philips Electronics). Day/night temperatures were 20°C and 15°C, respectively, and relative humidity was approximately 70%.

### Root-Zone-Specific, Subsampling-Based Analysis of Tracer Eﬄux

**FIGURE 1:**
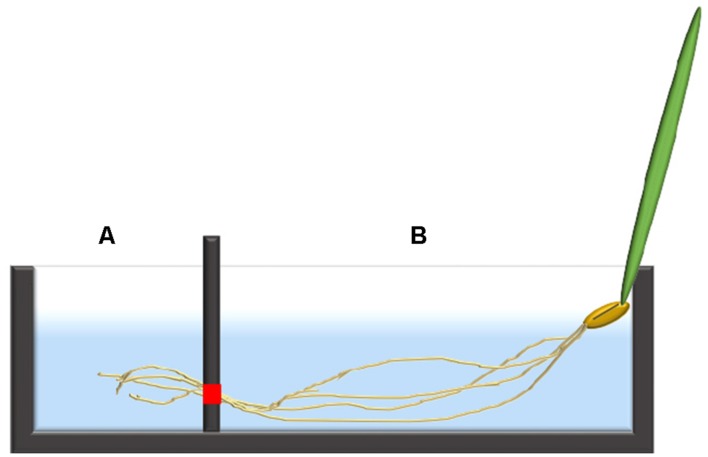
**An illustration of a single barley seedling fixed into the two-chambered cuvette system with the distal root in chamber (A) and the bulk root in chamber (B).** The red square indicates the location where the two pieces of the divider meet, allowing roots to pass through from chamber **(B)** into **(A)**, but otherwise sealed.

In both *Arabidopsis* and barley, the ^24^Na^+^-eﬄux protocol was partially based on a previously published tracer-subsampling protocol (Britto et al., 2006), coupled to the introduction of a two-chambered cuvette system (**Figure 1**). Single seedlings of 7-day-old barley, or three-plant bundles of 5-week-old *Arabidopsis* seedlings, were transferred into the custom-made acrylic uptake cuvettes. The average length of the roots were between 10–15 cm in barley and 20–25 cm in *Arabidopsis*. Each cuvette was divided into a chamber (“A”) containing the distal root region (i.e., typically 1–2 cm, 20 mL in volume), and a second chamber (“B”) containing the remainder of the root (“bulk root,” 30 mL in volume). The chambers were separated using a two-piece acrylic divider through which roots could pass, made watertight using vacuum sealant (Dow Corning high vacuum grease, Midland, MI, USA), to prevent solution leakage between chambers. Once sealed in place, the root tissue in both chambers was immersed for 1 h in a loading solution identical to the growth solution but containing ^24^Na^+^ (half-life of 14.96 h), received as NaCl from the McMaster University Nuclear Reactor (Hamilton, ON, Canada). After this loading step, the chambers were drained of radioactive solution, refilled with non-radioactive rinse solution (growth solution) for 5 s, and refilled again with growth solution for measurement of radioactivity, in the case of *Arabidopsis*. The technique was refined in the subsequent barley experiments, in which a second (5-min) rinse was introduced immediately after the first one, to reduce the high initial tracer background against which the ensuing tracer fluxes were difficult to discern. From the measuring solutions, 3-ml subsamples were periodically taken by pipette for gamma counting (Canberra-Packard Quantum Cobra Series II, Model 5003 γ-counter, Packard Instrument Co., Meriden, CT, USA), and replaced by the same volume of fresh measuring solution. These subsamples were taken from each chamber every 3 min for the first 15 min, then every 4 min for the remaining 20 min. In the pH 9.2 treatments, the measuring solution was as above, except that it was alkalinized using KOH.

Further details about the method are exposited in Section “Discussion.”

### Data Analysis

**FIGURE 2:**
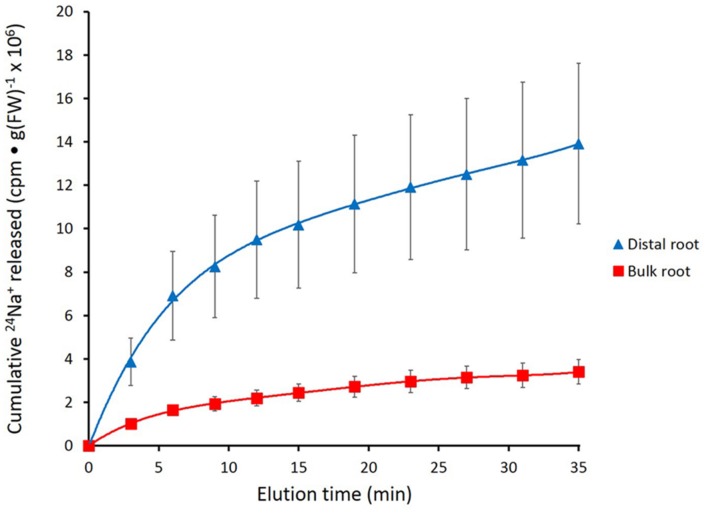
**Cumulative steady-state release of ^24^Na^+^ from the distal root and bulk roots of intact barley plants, labeled for 1 h in growth solution containing 100 mM NaCl.** Error bars indicate ± SEM (distal root *n* = 8; bulk root *n* = 7).

**FIGURE 3:**
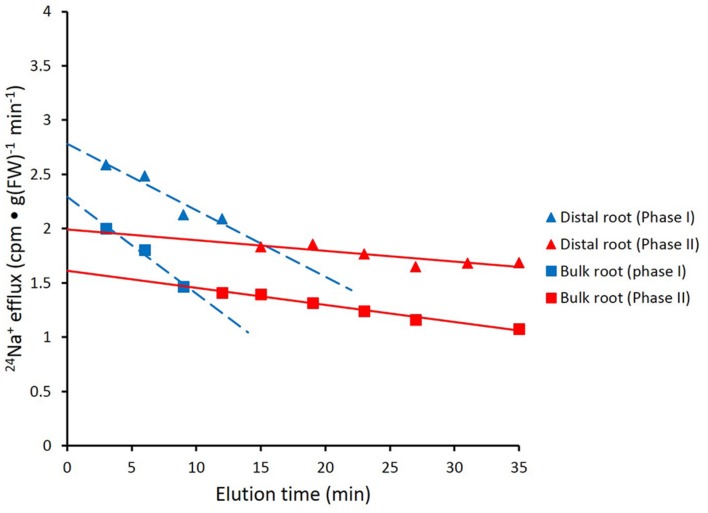
**Data from **Figure 2** recalculated to show biphasic tracer fluxes for the distal root and bulk roots of intact barley plants.** For linear coefficients, see **Table 1**.

**FIGURE 4:**
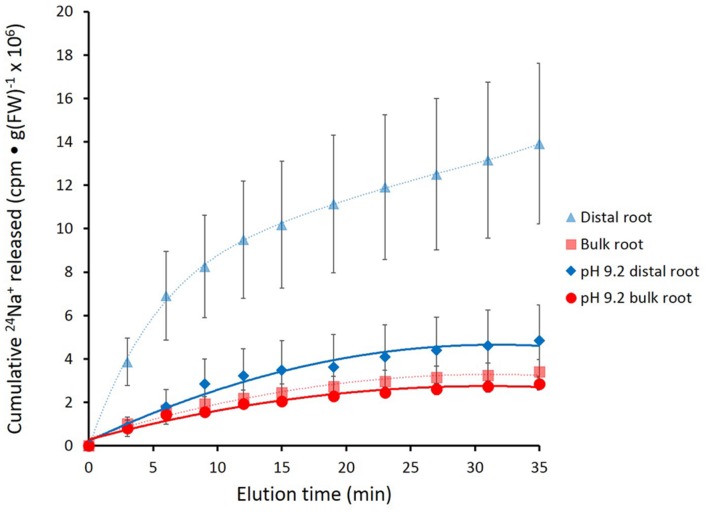
**Cumulative release of ^24^Na^+^ from the barley distal root and bulk roots, during exposure to elevated pH (9.2).** Control lines for each root zone are presented with dashed lines. Error bars indicate ± SEM (pH 9.2 distal root *n* = 6; pH 9.2 bulk root *n* = 6).

Cumulative cpm released by root zones (see **Figures 2**,**4**, and **6**) was calculated according to the formula given in Britto et al. (2006). To produce the release graphs, data were pooled from multiple experiments, and normalized to an arbitrary specific activity of 2 × 10^5^ cpm μmol^-1^. In some cases, as with barley under control conditions, it was possible to transform the cumulative release into a tracer flux, by dividing the increment of tracer released over a time interval by that interval (and the fresh weight of the root tissue). The changing tracer flux showed a compound exponential character (i.e., composed of additive functions of the form ϕ*_t__=_* ϕ*_i∗_e^-kt^*; see **Figure 3**), allowing treatment along the lines of classic compartmental analysis by tracer eﬄux (e.g., Walker and Pitman, 1976; Siddiqi et al., 1991).

## Results

**Figure 2** shows the cumulative steady-state release over time of ^24^Na^+^ from tracer-loaded distal root and bulk root of intact barley seedlings. At every sampling time, significantly more ^24^Na^+^ was released from the distal root than from the bulk root (*t*-tests comparing means of ^24^Na^+^ release at each time point, *p* < 0.05). In further analysis, it was possible to approximate the first derivatives of these curves, by calculating the changing tracer-eﬄux rates over the course of the experiments (**Figure 3**). Linear regression of these rates suggested that ^24^Na^+^ eﬄux proceeded in a biphasic pattern in both the distal root and bulk root, characterized by two exponentially declining components of tracer release (Phases I and II in **Figure 3**), and suggesting the involvement of two major tracer-releasing compartments. In **Table 1**, the rate constants (*k*-values) of these phases (and the corresponding half-times, *t½*), are given for each root zone. In addition, steady-state eﬄux from each compartment was estimated by extrapolation based on *k*-values and *y*-intercepts of the regressed lines, taking into account the estimated specific activities of each compartment, as well as the initial 5-min wash step used with barley (see Materials and Methods; Walker and Pitman, 1976; Siddiqi et al., 1991; Britto and Kronzucker, 2001). While the rate constants were fairly similar for a given phase in each root zone, the estimated eﬄux rates were five and three times higher (in Phases I and II, respectively) in the distal root compared to bulk roots. Eﬄux rates were also much higher in Phase I than Phase II in both root zones, reaching a maximum of nearly 2000 μmol g^-1^(FW) h^-1^ in Phase I of the distal root. Because of the short half-times and high maximum values of tracer release in Phase I, these exceptionally high fluxes were considered to be extracellular (see Discussion), consistent with previous compartmental analyses showing similarly high initial release rates (e.g., Kronzucker et al., 1995; Min et al., 1999).

**Table 1:** ^24^Na^+^-eﬄux parameters determined for root zones of barley plants grown and measured at 100 mM NaCl.

	Distal root		Bulk root	
	Phase I	Phase II	Phase I	Phase II
*k* (min^-1^)	0.225	0.0227	0.242	0.0363
*t½* (min)	3.08	30.5	2.86	19.1
eﬄux (μmol g^-1^(FW) h^-1^)	1920	148	446	46.1
*R*^2^	0.89	0.73	1^∗^	0.98

*^∗^Regression based only on two points.*

**FIGURE 5:**
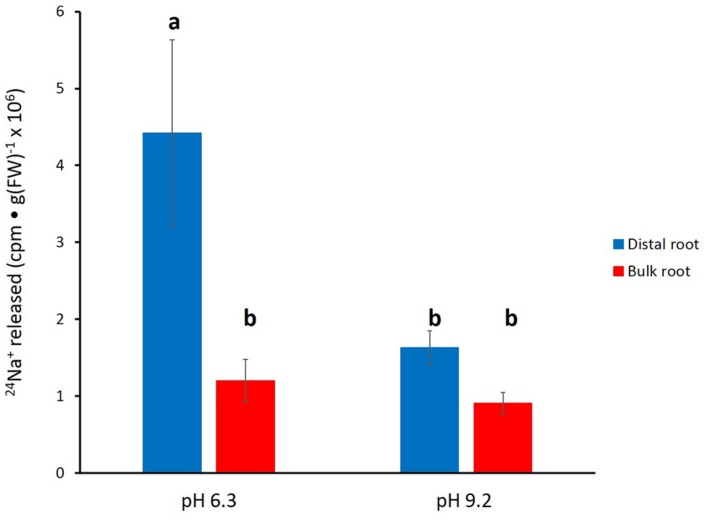
**Tracer released from the distal root and bulk roots of intact barley plants, following the first 12 min of elution (i.e., exclusively from Phase II).** Derived from data set in **Figure 4**. Error bars indicate ± SEM; *b* indicates significant differences from the control distal root *a* (*p* < 0.05, ANOVA followed by Holm multiple comparison test).

In **Figure 4**, the effects of alkaline (pH 9.2) conditions on ^24^Na^+^-release traces in barley are shown. Elevated pH brought about a pronounced reduction in tracer release from the distal root, but not in the bulk root. Unfortunately, resolution of the pH-response data into distinct phases of tracer eﬄux (as in **Figure 3**) was not possible due to the variability in the data (see Discussion). An alternative approach to quantitatively comparing the ^24^Na^+^-release patterns was to sum up the cpm released after the first 12 min of elution (i.e., over the course of exclusively Phase-II release from each root zone, essentially free of cpm released in Phase I), under control (pH 6.3–6.5) and alkaline conditions. Results from this procedure are shown in **Figure 5**, which indicates that tracer release from the control distal root was reduced by more than 60% by elevated pH, while there was no significant change in tracer release from the bulk root.

**FIGURE 6:**
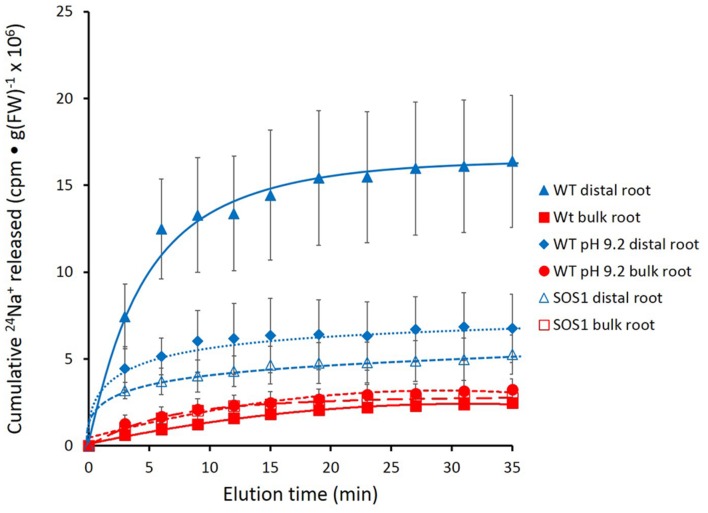
**Cumulative steady-state release of ^24^Na^+^ from the distal root and bulk roots of intact *Arabidopsis thaliana* plants (wild-type and *sos1*mutants), labeled for 1 h in growth solution containing 10 mM NaCl, and eluted at either pH 6 or pH 9.2.** Error bars indicate ± SEM (WT distal root *n* = 6; WT bulk root *n* = 6; WT pH 9.2 distal root *n* = 3; WT pH 9.2 bulk root *n* = 3; SOS1 distal root *n* = 5; SOS1 bulk root *n* = 5).

Further experimentation was pursued in *A. thaliana*, with particular focus on mutant plants lacking a functional *SOS1* gene. **Figure 6** shows that the roots of these plants released tracer in a manner similar to that seen in barley, with the largest fluxes being observed in the wild-type distal root under control conditions (pH 6). Tracer release in all other cases, i.e., in bulk roots of wild-type, in both the distal roots and bulk roots of *sos1* mutants, and in the distal roots and bulk roots wild-type plants exposed to alkaline (pH 9.2) treatments, was clearly lower than in the distal roots of control plants.

**FIGURE 7:**
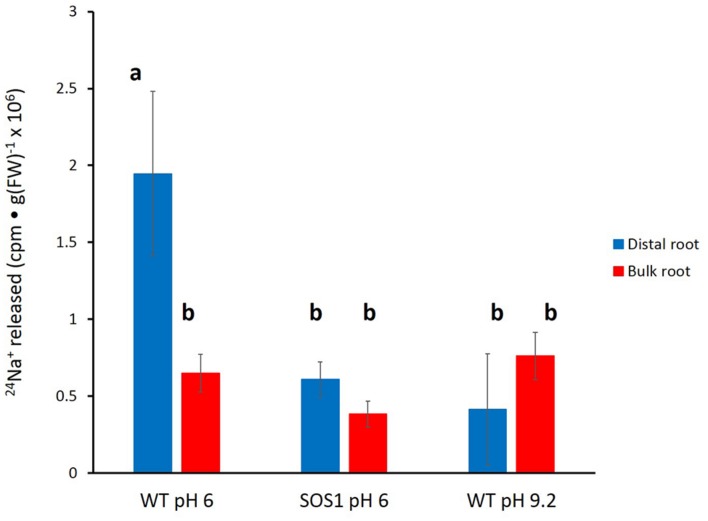
**Tracer released from the distal root and bulk roots of intact *Arabidopsis* plants (wild-type and *sos1* mutants), following the first 15 min of elution.** Derived from data set in **Figure 6**. Error bars indicate ± SEM; *b* indicates significant differences from the control distal root *a* (*p* < 0.05, ANOVA followed by Holm multiple comparison test).

Unfortunately, the variability in the data again did not allow for a clear resolution of the changes in tracer release over the course of the experiment. Thus, an approach similar to that taken for barley (**Figure 5**) was used for *A. thaliana*. Cumulative radioactivity released from the distal roots and bulk roots in the period beginning 15 min of elution is shown in **Figure 7**. As with barley, significantly (60–80%) more tracer was released by the distal root of wild-type plants (at pH 6) than from their bulk roots (pH 6 or 9.2), or from the *sos1* distal root and bulk roots (pH 6 or 9.2), or the wild-type distal root at pH 9.2. By contrast, tracer released by the distal root of *sos1* mutants was not significantly different from that released by *sos1* bulk roots, nor did pH treatment significantly affect tracer release from bulk roots of wild-type plants.

## Discussion

### Commentary on the Method

In this work, we have developed a system by which measurements of steady-state unidirectional ^24^Na^+^ eﬄux can be monitored in distinct regions of the plant root. Measurements of this nature are essential to an improved understanding of Na^+^ transport, because of the need to examine Na^+^ eﬄux in the context of the highly localized expression of SOS1, the only known Na^+^ eﬄux transporter in higher plants, in the distal root (as well as xylem parenchyma; Shi et al., 2002; see Britto and Kronzucker, 2015 for discussion). SOS1 expression and localization has been demonstrated in *A. thaliana* (Shi et al., 2002), but very few papers have shown SOS1 data in barley. However, Chen et al. (2015) showed that when barley plants were exposed to 150 mM NaCl for 48 h, gene expression of HvSOS1 in the roots increased significantly, as it does in *Arabidopsis.* This was the basis of our protocol involving Na^+^ pretreatment, which was applied to both species. Also, as mentioned above, the similarities in results between the two species suggest that SOS1 expression in barley is also likely to be at the distal root. The chambered-cuvette protocol we have established provides a clean separation between root zones, and also extends a subsampling method we developed earlier to examine whole-root fluxes with minimum perturbation due to handling (Britto et al., 2006; Malagoli et al., 2008).

While flux measurement in various root zones have been performed extensively using vibrating microelectrode systems such as MIFE and SIET (e.g., Sun et al., 2009; Cuin et al., 2011; Kong et al., 2012; Lang et al., 2014) these methods have generally been used only in the measurement of net, rather than unidirectional, fluxes, and even these are difficult or impossible to achieve in the presence of a large background of the substrate in question. Because this is the case with Na^+^-transport studies under salinity, Na^+^ eﬄux is typically not measured under steady-state conditions with these methods, but instead under conditions in which roots are initially Na^+^-loaded at high external [Na^+^] ([Na^+^]_ext_, typically ≥ 50 mM), and subsequently measured under low [Na^+^]_ext_ (Britto and Kronzucker, 2015). While this approach has provided some interesting findings (see below), it entails a very large change in the electrochemical potential gradient for Na^+^ across the plasma membrane of the root cells, and therefore cannot provide information about the Na^+^ transport cycle under steady-state salinity conditions (see Introduction; Britto and Kronzucker, 2015).

While the use of radiotracers in the present study, by contrast, allows for the measuring of unidirectional fluxes of Na^+^ (and, by extension, other ions) under steady-state salinity conditions, it is not without some difficulties of its own.

First, it is very important that the plants are handled with great care and that the roots, especially the distal roots, are not damaged while the distal roots are separated from the remainder of the root and these zones are isolated into their respective chambers. When working with *Arabidopsis* in particular, the roots are very fine and fragile, and the risk of damage is higher than with barley roots, which are larger and easier to separate and handle. In addition, it is difficult to separate the individual *Arabidopsis* roots from one another and ensure that tissue from a particular root zone does not become sealed in the wrong chamber. This is in part because *Arabidopsis* also has many secondary roots branching along the entire primary root axis, which may include SOS1 transporters at their distal root. This is not a pronounced issue with barley seedlings, which more easily permit a cleaner separation of the distal root from the rest of the root. It should be noted that difficulties with the application of radiotracer technology to *Arabidopsis* seedlings have been discussed by others (e.g., Elphick et al., 2001).

Another major obstacle to be overcome with the chambered-cuvette system was the potential for leakage between adjacent chambers. In order to minimize leakage, high-vacuum sealant was applied around to the single area of communication between the two chambers, i.e., where the roots traverse from one chamber to the next. Because barley roots are much larger in diameter than *Arabidopsis* roots, the potential for leakage was higher and therefore much more care was required. With barley, each root had to be individually placed on a layer of sealant, sufficiently far from other individual roots, to avoid small gaps in the seal and ensure that no leakage occurs. To test the seal, plants were introduced into the cuvette and radioactive solution was applied to one chamber, but not the other. The other chamber was then monitored for radioactivity. The results showed that, with care, it was possible to achieve no detectable leakage from one chamber to the other (not shown). Because barley roots are large, and the potential for leakage was higher, we only used one seedling per chamber per experimental run (by contrast, bundles of three *Arabidopsis* plants were used), which was likely responsible for some of the variability in the barley data.

Another issue that arose when separating the two root zones was the necessity of ensuring that the distal root chamber contained enough root material such that it could be weighed accurately. This was especially problematic with *Arabidopsis* because the roots are very fine and have little mass. The more root material that is introduced into the distal root chamber, however, the more likely it is that there will be a significant amount of bulk root in that same chamber. Thus, a compromise between having a measurable mass on the one hand, and as little bulk root as possible on the other, must be struck using the present protocol.

In the initial trials, the above issues (mostly leakage) led to the discarding of some 75% of poor-quality runs. With practice, taking the precautions outlined above, we found that this number could be reduced to about one-third.

### Discussion of Results

Despite the difficulties associated with the method, we were able to produce enough high-quality replicates with which to draw valuable conclusions about sodium eﬄux in barley and *Arabidopsis*. In particular, we found that the distal root had at least 3–4 times the eﬄux activity of bulk roots in both species (**Figures 2–7**), confirming a hypothesis put forward in our recent review on Na^+^ eﬄux in higher plants (Britto and Kronzucker, 2015; also see Introduction). This finding is a physiological validation of the role of SOS1 in Na^+^ eﬄux, given that the protein is highly expressed only in the distal root (and in vascular tissue) but not in epidermal or cortical tissues of the bulk root (Shi et al., 2002). It has been proposed by others that SOS1 expression in distal root cells is directly related to the lack of vacuolation in these cells (Shi et al., 2002; Oh et al., 2009); thus, excess sodium entering across the plasma membrane cannot be sequestered via tonoplast transport via the NHX1 protein (Apse et al., 1999), but must be expelled via SOS1. This localized role has been previously suggested in vibrating microelectrode studies, where measurements are often preferably made at the distal root because this is where a “vigorous flux” is generally seen (e.g., Kong et al., 2012; Lang et al., 2014, and references therein). However, as noted above, this method cannot be used for the measurement of steady-state, unidirectional Na^+^ eﬄux, but only for net eﬄux of Na^+^ proceeding after a large reduction of external [Na^+^] and, thus, a shift in the electrochemical potential gradient for the ion.

We have shown here that Na^+^ eﬄux from the distal root of both plant species is strongly suppressed by elevated pH, while eﬄux from bulk roots was not significantly affected (**Figures 4–7**). This further supports a specific role of SOS1 in the distal root, given that it has been shown that SOS1 operates as a Na^+^/H^+^ antiporter, using fluorescent probes in plasma-membrane vesicles (Qiu et al., 2002). Given this mechanism, it is surprising that almost no *in planta* work has been conducted to investigate the pH-dependence of Na^+^ fluxes; an exception is the decades-old study by Ratner and Jacoby (1976), which showed a reversible high-pH-induced suppression of Na^+^ release in distal root segments of barley. However, this was conducted at an external [Na^+^] of 20 mM (vs. 100 mM in the present study), not a stress condition in this relatively salt-tolerant species.

A third finding to support the role of SOS1 in our study was the pronounced suppression of Na^+^ eﬄux in the distal root of *sos1* mutants of *Arabidopsis* plants relative to wild-type, but no change in the pattern of ^24^Na^+^ release from bulk roots of this genotype. T his finding is significant in that, to our knowledge, no direct *in planta* demonstration of the unidirectional Na^+^-eﬄux function of SOS1 has until now come forward, although this function has long been inferred from indirect evidence such as net-flux measurements (e.g., Cuin et al., 2011). Moreover, ours is the first demonstration of this kind made using radiotracer (see Britto and Kronzucker, 2015), although ^22^Na^+^ eﬄux was shown to be suppressed in *Arabidopsis* deficient in the gene encoding SOS3, which is involved in the calcium-dependent activation of SOS1 (Elphick et al., 2001).

While the physiological significance of our results is clear, the ^24^Na^+^ traces in both barley and *Arabidopsis* present a difficult problem. The very rapid initial release of tracer (i.e., in the first 5 min), was considered to be extracellular because of the magnitude of release and the short half-times as quantified in barley (**Table 1**). However, the experiments show that, in both species, elevated pH greatly reduces this initial flux, and, moreover, in *Arabidopsis*, the *sos1* mutation also results in a great reduction (**Figures 4** and **6**). While this enigma cannot be firmly resolved at present, it may be reflective of pH-dependent cation binding processes in the carbohydrate- and protein-rich root cap area (Baetz and Martinoia, 2014) on the one hand, and the numerous non-transport functions (pleiotropies?) that have been related or attributed to the SOS1 protein (Sun et al., 2008; Oh et al., 2009; Ji et al., 2013; Britto and Kronzucker, 2015).

A related question about compartment identity can be raised regarding the nature of the tracer-releasing pool in the bulk root zone of both species. This zone is affected neither by pH treatment nor by *sos1* mutation, yet still appears to be responsible for a substantial flux (**Figures 4** and **7**), which in a first approximation was calculated to be about 46 μmol g^-1^(FW) h^-1^ (**Table 1**) in barley at 100 mM. We have previously argued that large values for ionic eﬄux (and, hence, influx) from plant

roots at high external concentrations may be largely due to tracer flowing through the root apoplast (including in the slower phases; Britto et al., 2010; Britto and Kronzucker, 2015; Coskun et al., 2015), and suggest that the bulk root fluxes reported here may be of this nature. Our argument, however, rests in large part upon the very low degree, or absence, of Na^+^-eﬄux malleability when inhibitory treatments have been applied (Britto and Kronzucker, 2015, and references therein). However, the malleability of the initial, putatively apoplastic phase of tracer release both by elevated pH and *sos1* mutation (**Figures 4** and **6**) suggest that neither condition presents an adequate parsing tool to distinguish apoplastic from symplastic tracer eﬄux, and more trenchant experimentation will need to be applied to resolve this issue.

Lastly, it is worth considering the even higher value of ^24^Na^+^ eﬄux from the slow phase in the distal root, in terms of the energy that would be required to drive this thermodynamically active process. We have estimated this flux to be 148 μmol g^-1^(FW) h^-1^ in barley at 100 mM NaCl (**Table 1**), which would entail a very large respiratory burden on root cells were it to be entirely a symplastic flux (Britto and Kronzucker, 2009, 2015). However, it is known that distal-root cells have much higher respiratory activity than cells in the remainder of the root (e.g., Luxmoore and Stolzy, 1972). In addition, some fraction of this flux may yet be apoplastic, given that elevated pH and the *sos1* mutation appear to reduce the initial (apoplastic) phase of tracer release. Regardless of these considerations, the malleability of the distal root eﬄux system(s) is not likely to be observed in standard eﬄux experiments involving the whole root, given that the mass of the distal root is very small in relation to the bulk root. Given that the mass of the distal root was less than 5% in barley and less than 1% in *Arabidopsis*, in the present study, the contribution of the distal root to the overall Na^+^ eﬄux is only 16% in barley and smaller than 1% in both *Arabidopsis* WT and *sos1* mutant.

## Conclusion and Future Directions

The method described in this paper is relatively straightforward in execution, but sheds new insight into Na^+^ eﬄux from plant roots, is adaptable to taxonomically distant plant species, and promises to allow further discovery. While the strong localization of SOS1 expression in the distal root, as well as non-steady-state net flux measurements using ion-selective external microelectrodes, have provided early indications that the distal root may be a zone of intense sodium export from within cells, ours is the first demonstration, and comparison, of steady-state, unidirectional fluxes from this critical root zone and the bulk root. By isolating the distal root, we can further investigate the physiology of SOS1-mediated Na^+^ eﬄux from roots, to better resolve the capacity of this system, and its sensitivities (e.g., to other pH treatments, to inhibitors such as amiloride, to genetic conditions such as *sos2* and *sos3* mutations, and to the induction of the Na^+^ eﬄux system by external Na^+^ and Ca^2+^).

We think that the physiological questions that can be addressed using this technique are significant enough to justify further development of the protocol, as they may help make progress toward the elusive goal of identifying and characterizing sodium transport in plant roots in the saline range, and the even more elusive goal of improving plant salt tolerance by the modification of the transport systems involved.

## Author Contributions

Each of the four authors have contributed a significant amount of work to the paper. AH was heavily involved in experimental design and execution, data analysis, as well as manuscript preparation and writing. DB was largely involved in experimental design, data analysis, and manuscript preparation and writing. RF-S contributed mainly to experimental design and execution. HK oversaw all aspects of the work, and was heavily involved in the experimental design, data analysis, and manuscript preparation.

## Conflict of Interest Statement

The authors declare that the research was conducted in the absence of any commercial or financial relationships that could be construed as a potential conflict of interest. The reviewer KY and handling Editor declared their shared affiliation, and the handling Editor states that the process nevertheless met the standards of a fair and objective review.
